# No Immediate Effects of Transcranial Direct Current Stimulation at Various Intensities on Cerebral Blood Flow in People with Multiple Sclerosis

**DOI:** 10.3390/brainsci10020082

**Published:** 2020-02-04

**Authors:** Craig D. Workman, Laura L. Boles Ponto, John Kamholz, Thorsten Rudroff

**Affiliations:** 1 Department of Health and Human Physiology, University of Iowa, Iowa City, IA 52242, USA; craig-workman@uiowa.edu; 2 Department of Radiology, University of Iowa Hospitals and Clinics, Iowa City, IA 52242, USA; laura-ponto@uiowa.edu; 3 Department of Neurology, University of Iowa Hospitals and Clinics, Iowa City, IA 52242, USA; john-kamholz@uiowa.edu

**Keywords:** tDCS, neuroimaging, positron emission tomography, cerebral blood flow, multiple sclerosis

## Abstract

Animal and transcranial magnetic stimulation motors have evoked potential studies suggesting that the currently used transcranial direct current stimulation (tDCS) intensities produce measurable physiological changes. However, the validity, mechanisms, and general efficacy of this stimulation modality are currently being scrutinized. The purpose of this pilot study was to investigate the effects of dorsolateral prefrontal cortex tDCS on cerebral blood flow. A sample of three people with multiple sclerosis underwent two blocks of five randomly assigned tDCS intensities (1, 2, 3, 4 mA, and sham; 5 min each) and [^15^O]water positron emission tomography imaging. The relative regional (i.e., areas under the electrodes) and global cerebral blood flow were calculated. The results revealed no notable differences in regional or global cerebral blood flow from the different tDCS intensities. Thus, 5 min of tDCS at 1, 2, 3, and 4 mA did not result in immediate changes in cerebral blood flow. To achieve sufficient magnitudes of intracranial electrical fields without direct peripheral side effects, novel methods may be required.

## 1. Introduction

Multiple sclerosis (MS) is a chronic central nervous system disease that affects approximately 2.3 million people worldwide [1]. Because some MS symptoms (e.g., neuropathic pain) are treatment-resistant [2], practical and inexpensive adjunctive therapies, like transcranial direct current stimulation (tDCS), are of high interest. Despite promising findings in tDCS studies in people with multiple sclerosis (PwMS) [3,4,5] (see [6] for a review), the validity and utility of tDCS is under scrutiny. For example, a critical review [7] did not support the idea that tDCS has a reliable neurophysiological effect beyond motor evoked potential (MEP) amplitude modulation. Though MEP amplitude appears to be sensitive to tDCS modulation, other reliable transcranial magnetic stimulation (TMS) measures that rely on similar neural mechanisms (e.g., short interval intracortical inhibition (SICI), intracortical facilitation (ICF), and cortical silent period (cSP)) have all shown no tDCS effect. Questions concerning the mechanistic foundations and general efficacy of this stimulation modality are on the rise.

Animal studies have suggested that the intensities that are typically employed in human research are sufficient to produce measurable physiological changes [8]. Unfortunately, findings from animal models, especially in vitro approaches, may have poor applicability to human studies. Thus, a vital concern is that tDCS may not induce a sufficient current in the cortex to have a measurable effect on neural function—at least not for the commonly used stimulation intensities (≤2 mA). Therefore, it is necessary to combine tDCS with human neuroimaging to complement animal studies and to further clarify whether tDCS can affect neural function.

Cerebral activity in PwMS has been investigated with [^15^O]water [9]. A close coupling of perfusion and metabolism was assumed, as this reflects the oxidative phosphorylation of glucose as the predominant energy source. Consequently, cerebral blood flow (CBF) is often considered an indirect measure of neuronal function and integrity [10]. This is supported by the significant association of glucose metabolism with regional CBF (rCBF) across different brain regions and global CBF (gCBF) across varying states of consciousness.

## 2. Materials and Methods

**Figure 1:**
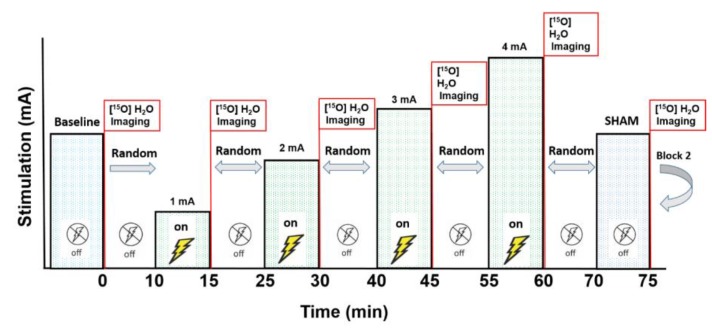
The stimulation and scan protocol. After the baseline scan, the five stimulation intensities (1, 2, 3, 4 mA, and sham) were delivered for 5 min each in a random order (six total scans per block). Ten minutes after the final scan of Block 1, Block 2 began with a new baseline scan and a different intensity randomization. Each subject experienced 12 total scans.

We conducted a pilot investigation on the effects of tDCS on CBF with semi-quantitative [^15^O]water Positron Emission Tomography (PET), the gold standard for CBF measurements [11,12,13,14,15], in a sample of three people with relapsing–remitting MS (Table 1). Scans were completed in two blocks, one at baseline and five immediately after randomized tDCS stimulation intensities (1, 2, 3, 4 mA, and sham; six scans per block). The second block (intensity re-randomized) helped verify the reliability of the first. Therefore, each subject experienced 12 total scans in one session (Figure 1). The subjects performed a simple counting task for the duration of each scan (100 sec). This study was approved by the University of Iowa’s Institutional Review Board (IRB-01: IRB#201905826; clinicaltrials.gov NCT04033133). All subjects provided written informed consent before participating.

A battery-operated tDCS device (Soterix Medical Inc., New York, NY, USA) administered the stimulation. A previous study indicated that 5 min of tDCS over the primary motor cortex (M1) was sufficient to induce significant increases in MEP amplitude and that the effects of the stimulation returned to baseline after 10 min [16], and another study indicated significant blood flow changes from 4 min of motor cortex stimulation [17]. Thus, the tDCS parameters in this study included 5 min of stimulation at each intensity, each separated by ≥10 min, to avoid stimulation carry-over effects; this also allowed for sufficient time for the [^15^O]water tracer to decay before the next injection. The stimulation was ramped up to the target intensity (1, 2, 3, or 4 mA) over 30 s, after which time the intensity was maintained for 5 min before ramping down to 0 mA over 30 s. The anode was over the left dorsolateral prefrontal cortex (dlPFC), and the cathode was over the contralateral supraorbital area. A dlPFC target helped to avoid potentially confounding M1 activity from counting or spontaneous movement during scanning. 

Because significant excitability increases from 5 min of tDCS have been found at 1 min post-tDCS [16], imaging commenced 1 min after the ramp-down process was completed. A summed image of the 40 seconds immediate post-bolus transit was generated for each scan. The summed images were co-registered with the subject’s T1-weighted magnetic resonance imaging (MRI). Individual, anatomically-based regions were defined by using the PNEURO tool of the PMOD Biomedical Image Quantification software package (PMOD Technologies, Ltd. Zürich, Switzerland. The mean global activity (gCBF) was calculated based on the volume-weighted average of all intracerebral regions. The CBF relative to the global activity was calculated for each region (rCBF = regional activity/gCBF; e.g., ratio 1.2 = rCBF 20% higher than gCBF) for each condition. Changes in gCBF and rCBF, focusing on regions under the electrodes, at the different tDCS intensities were investigated.

## 3. Results

**Figure 2:**
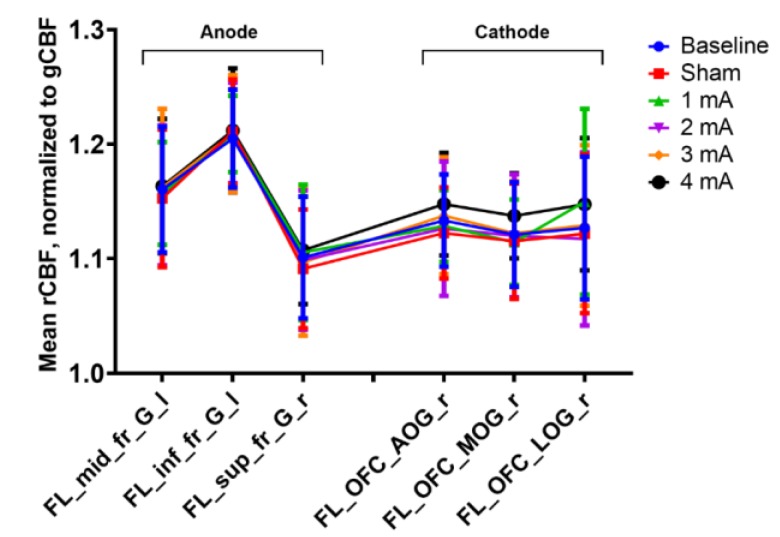
Mean tracer uptake (regional cerebral blood flow (rCBF)) relative to global tracer uptake (global CBF (gCBF)) by region and condition. Data are mean ± SEM. FL_mid_fr_G_l = left middle frontal gyrus, FL_inf_fr_G_l = left inferior frontal gyrus, FL_sup_fr_G_l = left superior frontal gyrus, FL_OFC_AOG_r = right anterior orbital gyrus, FL_OFC_MOG_r = right medial orbital gyrus, and FL_OFC_LOG_r = right lateral orbital gyrus.

The results revealed no notable differences in the gCBF or rCBF for the areas under the electrodes from the different tDCS intensities (Figure 2). We did not find any immediate effects (i.e., ~1–2 min post-stimulation) of tDCS on CBF in this functional [^15^O]water PET study. None of the stimulation intensities (1, 2, 3, and 4 mA) over dlPFC were associated with changes in gCBF (not shown) or rCBF under the electrodes.

## 4. Discussion

There are several possible explanations for this finding: (1) tDCS may not induce a sufficient current in the cortex to have a measurable effect on neural function. Vöröslakos et al. [18] suggested that a 1 V/m minimum voltage gradient was required for measurable online stimulation effects on neuronal spiking or membrane potentials in a rat model. To achieve 1 V/m at the cortex, the current applied at the scalp may need to be as high as 4–6 mA; however, the 4 mA that was used in this study did not elicit any CBF changes. (2) Only a small fraction of the transcranial current may reach the brain. One study [18] in rodents and human cadavers demonstrated that >75% of applied current is lost at the scalp, subcutaneous tissue, and muscle. These tissues serve as an effective shunt, resulting in at least a 50% reduction of current intensity. In addition, the serial resistance of the skull further reduces the current flow by 10%–25%, depending on skull thickness [18]. (3) Inter-individual variability in tDCS, with approximately 50% of people having poor or absent responses [19], may have biased our results. (4) Previous neuroimaging studies on the neurophysiological effects of tDCS detected immediate effects of tDCS on CBF by using [^15^O]water PET [17,20]. However, these studies targeted M1 during a motor task, and diverse tDCS effects on different brain regions cannot be excluded. (5) Stimulating nerves on the scalp could also send signals to the brain or influence circulation, and/or other non-neuronal cells may react to the induced electrical fields and gradually alter brain function. Lastly, (6) the significant findings reported in the tDCS literature have assumed to have reflected one or more of a number of factors including placebo effects, influences from peripheral nerve stimulation, poor experimental designs, low statistical power, or inappropriate control conditions or analyses. 

The sample size of this pilot study was small, and more subjects are recommended to confirm or refute these results. However, the study design, which included 12 injections that encompassed all conditions in duplicate, provided robust and remarkably consistent information, both between conditions and between subjects. Additionally, given that PET studies are costly and exposing the fewest subjects as possible to radiation is a major consideration [21], collecting images from additional subjects when there were no evident effects in these three PwMS was not justified. Furthermore, one of the purposes of reports like the present study is to encourage discussion and highlight the need to confirm the mechanistic effects of tDCS with neuroimaging (e.g., [^15^O]water PET, [^18^F]-fluorodeoxyglucose PET, functional magnetic resonance imaging (fMRI), and arterial spin labelling (ASL)). It should also be mentioned that multiple interventions over short periods might have complex and non-linear effects on the brain. Thus, any effect (or lack of effect) should to be interpreted with caution. Additionally, the absence of immediate tDCS effects on the cortex does not preclude longer-term effects. Furthermore, these findings were in a sample of PwMS, and different effects in healthy or patient populations cannot be excluded. Finally, this is a growing field for research, and a comprehensive understanding of how tDCS modulates the cortex is necessary. In humans, physiological changes associated with tDCS at different intensities and durations can be assessed with a variety of different neuroimaging methods (e.g., PET, fMRI, and ASL). All of these techniques have their limitations, but [^15^O]water PET is considered to be the most “direct” measurement of cerebral blood flow. Thus, routinely combining neuroimaging with tDCS will help provide the necessary evidence that tDCS can affect neural function in humans.

## 5. Conclusions

In conclusion, we have demonstrated that 5 min of dlPFC tDCS at 1, 2, 3, or 4 mA did not result in immediate changes in CBF in PwMS. Thus, to achieve sufficient magnitudes of intracranial electrical fields without direct peripheral side effects, novel methods may be required. 

## Figures and Tables

**Table 1:** Subject demographic information (*n* = 3). Data are mean ± SD.

Sex (M/F)	2/1
Age (years)	45.3 ± 19.0
Height (cm)	171.9 ± 18.7
Weight (kg)	83.5 ± 19.9
Time since diagnosis (years)	8.0 ± 5.3
Patient-Determined Disease Steps ^1^	2.3 ± 2.1

^1^ Provides an indication of disease severity. A score of 2–4 out of 8 indicates moderate disability.
